# Molecular Characterization of Pathogenic Avian Reovirus Circulating in Clinically Affected Chickens in Southeastern China (2022–2023) and Its Immunosuppressive Interference with Fowl Adenovirus Serotype 4 Vaccination

**DOI:** 10.3390/microorganisms14030676

**Published:** 2026-03-16

**Authors:** Xiaojian Chen, Yazheng Chen, Shenghua Yang, Yanhua Xu, Xuesong Li, Zhanxin Wang, Lijuan Yin, Wencheng Lin

**Affiliations:** 1 College of Animal Science, South China Agricultural University, Guangzhou 510642, China; 2 Wen’s Foodstuffs Group Co., Ltd., Yunfu 527400, Chinaxyh131625@163.com (Y.X.); wangzhanxin1985@163.com (Z.W.); 3 Zhaoqing Academy of Agricultural and Forestry Sciences, Zhaoqing 526061, China; 4 Yunfu Branch, Guangdong Laboratory for Lingnan Modern Agriculture, Yunfu 527400, China

**Keywords:** avian reovirus, molecular epidemiology, immunosuppression, fowl adenovirus serotype 4, vaccine interference

## Abstract

Avian reovirus (ARV) is a ubiquitous pathogen in commercial poultry, traditionally associated with viral arthritis, malabsorption syndrome, and growth retardation. In recent years, the rapid genetic diversification of ARV has raised increasing concerns regarding vaccine mismatch, immune dysregulation, and complex disease outcomes in vaccinated flocks. In this study, an integrated investigation combining large-scale field surveillance, molecular characterization, and controlled animal experiments was conducted to elucidate the epidemiological features of ARV and its impact on heterologous vaccine-induced protection. Epidemiological surveillance revealed widespread ARV circulation in commercial poultry flocks, with marked genetic divergence between contemporary field isolates and classical vaccine strains. Phylogenetic analysis based on the *σC* gene demonstrated that the majority of circulating strains clustered within emerging genotypes that were genetically distinct from vaccine-related lineages. Using a controlled infection–vaccination–challenge model, prior ARV infection was shown to significantly impair humoral immune responses induced by an inactivated *Aviadenovirus hydropericardii* (fowl adenovirus serotype 4, FAdV-4) vaccine, as evidenced by reduced FAdV-4-specific antibody levels. Importantly, ARV pre-infection compromised vaccine-mediated protection and was associated with enhanced FAdV-4 pathogenicity following challenge, resulting in increased mortality, aggravated clinical manifestations, and more pronounced pathological lesions. These findings indicate that prior ARV infection is associated with reduced FAdV-4 vaccine-induced humoral responses and partial loss of protective efficacy under controlled experimental conditions. Importantly, this study provides quantitative experimental evidence using a defined infection–vaccination–challenge interference model rather than proposing a previously unrecognized virus-virus interaction. These results underscore the necessity of enhanced ARV surveillance and optimized immunization strategies in modern poultry production systems.

## 1. Introduction

Avian reovirus (ARV) is a genetically diverse, non-enveloped, double-stranded RNA virus belonging to the genus *orthoreovirus* within the family *reoviridae* and is ubiquitous in poultry production systems worldwide [1,2]. The viral S1 genome segment encodes the σC protein, which is responsible for mediating viral attachment to host cells and contains both highly variable and conserved antigenic domains [3]. Sequence analysis of the *σC* gene has therefore been widely applied as a practical approach for molecular epidemiological surveillance and preliminary genotypic classification of ARV strains. However, this strategy does not fully capture whole-genome relatedness or reassortment events, which are common features of segmented RNA viruses [4]. In addition, the complex multilayered capsid architecture of ARV confers marked resistance to environmental stresses, thereby enhancing viral persistence and transmission within commercial poultry flocks [2]. Phylogenetic analyses have demonstrated that circulating ARV strains can be grouped into at least six distinct genotypes, reflecting substantial genetic heterogeneity [5,6]. This extensive genetic diversity is closely associated with pronounced antigenic variation, which contributes to the wide range of clinical manifestations observed following ARV infection [2]. Historically, ARV infection has been associated with a spectrum of clinical syndromes, including viral arthritis/tenosynovitis, runting-stunting syndrome, and enteric disorders, which collectively compromise flock health, growth performance, and production efficiency [2,7,8].

Despite the widespread use of classical ARV vaccines in breeder and broiler operations, disease control is increasingly challenged by the continuous emergence of genetically and antigenically divergent variant strains [9,10]. In recent years, clinically significant outbreaks have been repeatedly reported in well-vaccinated flocks, particularly those characterized by viral arthritis and growth retardation, suggesting inadequate cross-protective immunity between vaccine strains and circulating field isolates [11,12]. These epidemiological observations underscore the dynamic evolution of ARV and highlight the urgent need to characterize the molecular diversity of contemporary ARV strains [13].

ARV has also been implicated in modulation of host immune responses and is frequently associated with reduced vaccine performance under both field and experimental conditions [14]. Field investigations frequently co-detect ARV with other immunosuppressive pathogens, including infectious bursal disease virus (IBDV), and chicken infectious anemia virus (CIAV), implicating ARV as a key component of multifactorial immunosuppressive complexes in poultry [15]. In addition to IBDV and CIAV, a wide range of infectious, toxic, and environmental factors are recognized contributors to immune dysfunction in chickens. These include viral agents such as Marek’s disease virus and fowl adenoviruses, mycotoxin exposure (e.g., aflatoxicosis), and non-infectious stressors such as high stocking density, inadequate nutrition or water availability, elevated ammonia concentrations, and poor ventilation [16]. Collectively, these factors may interact to compromise immune competence and reduce the effectiveness of vaccination programs in intensive poultry production systems. Experimentally, ARV infection induces lymphoid organ atrophy, suppresses lymphocyte proliferation, and disrupts cytokine and interferon signaling pathways [17,18]. Notably, the ARV σA protein antagonizes type I interferon responses by inhibiting interferon regulatory factor 7 (IRF7) activation, providing a mechanistic basis for virus-mediated immune evasion and immunosuppression [19].

*Aviadenovirus hydropericardii* (fowl adenovirus serotype 4, FAdV-4), the causative agent of hepatitis-hydropericardium syndrome (HHS), is one of the most economically devastating viral diseases in contemporary poultry production, particularly affecting broiler chickens and breeder flocks [20,21]. Infection with hypervirulent FAdV-4 strains is characterized by high mortality, severe hepatic necrosis, hydropericardium, and substantial economic losses [22]. Accordingly, vaccination is the cornerstone of control, with multiple inactivated, subunit, and recombinant vaccines developed and implemented [23,24,25]. Nevertheless, inconsistent vaccine performance and sporadic outbreaks persist, suggesting that host-related factors critically influence protective efficacy [22].

Accumulating evidence indicates that host immune status and concurrent immunosuppressive infections play a decisive role in shaping vaccine-induced protection. Co-infections with immunosuppressive viruses can impair humoral and cellular immune responses, thereby reducing heterologous vaccine efficacy and exacerbating disease severity. In the context of FAdV-4, synergistic pathogenic interactions with immunosuppressive viruses such as IBDV and ARV have been increasingly reported, representing a “hidden threat” to vaccination-based control [26,27,28,29]. Notably, infection with novel variant IBDV significantly diminishes FAdV-4 vaccine efficacy and enhances pathogenicity, supporting a framework whereby virus-induced immune dysregulation contributes to suboptimal vaccine performance and aggravated disease [26,27].

The ARV–FAdV-4 interaction model was selected in this study based on both biological and epidemiological considerations. In recent years, FAdV-4-associated HHS has re-emerged as a major cause of economic losses in Chinese poultry production, despite the widespread use of inactivated vaccines. Field investigations have increasingly reported FAdV-4 outbreaks in flocks with evidence of early ARV circulation, raising concerns regarding potential vaccine interference rather than complete vaccine failure. From an immunological perspective, protective immunity against FAdV-4 relies predominantly on effective humoral responses elicited by inactivated vaccines, which may be particularly sensitive to early immune perturbations. Accordingly, FAdV-4 provides a biologically relevant and sensitive model to evaluate whether prior ARV infection can directionally impair heterologous vaccine-induced protection under controlled experimental conditions.

Despite the widespread prevalence and documented immunosuppressive potential of ARV, comprehensive investigations integrating molecular epidemiology with experimental assessment of its immune interference are scarce. In particular, although ARV-FAdV interactions have been reported previously, the quantitative impact of ARV infection on FAdV-4 vaccine-induced humoral immunity and protective efficacy has not been systematically evaluated using a controlled infection- vaccination-challenge framework. Therefore, the present study aimed to (i) investigate the prevalence and genetic diversity of ARV circulating in commercial poultry flocks, (ii) characterize the molecular features of contemporary ARV strains, and (iii) assess the effects of prior ARV infection on FAdV-4 vaccine-induced antibody responses, protective efficacy, viral replication, and pathological outcomes following challenge. In this study, immune impairment is evaluated primarily at the level of vaccine-induced humoral responses and protection, rather than through comprehensive immunological profiling. Collectively, these findings provide insight into ARV-associated immune interference and offer an evidence-based framework for optimizing integrated vaccination and disease control strategies in poultry production systems.

## 2. Materials and Methods

### 2.1. Ethical Statement

All animal experiments were reviewed and approved by the Animal Care Committee of South China Agricultural University (approval ID: SYXK-2024-0136). All experimental procedures were conducted in strict accordance with the Guidelines for the Care and Use of Laboratory Animals issued by the Ministry of Science and Technology of the People’s Republic of China. To ensure adequate anesthesia and minimize pain and distress, birds were deeply anesthetized with isoflurane delivered in oxygen, followed by euthanasia via parenteral administration of pentobarbitone, in accordance with a previously validated protocol for stable and humane anesthesia in broiler chickens [30].

### 2.2. Animals, Viruses, and Vaccine

All specific-pathogen-free (SPF) chicken embryos and SPF chickens were purchased from the SPF Experimental Animal Center of Guangdong Dahuanong Poultry and Egg Products Co., Ltd. (Yunfu, Guangdong, China). Birds were housed in negative-pressure isolators under biosafety conditions with ad libitum access to feed and water.

The virulent *Aviadenovirus hydropericardii* (fowl adenovirus serotype 4, FAdV-4) strain HN1605 was originally isolated from a broiler chicken flock experiencing an outbreak of hydropericardium-hepatitis syndrome (HHS) in Henan Province, China, The HN1605 strain has been previously characterized as highly pathogenic [26]. The strain has been maintained in our laboratory with limited passage history prior to experimental use to minimize potential attenuation.

The ARV strain GX-742-2022 was isolated from the gastrocnemius tendon and synovial tissues of a commercial broiler chicken flock with an outbreak characterized by clinical tenosynovitis and lameness in Guangxi Province, China. All the affected birds exhibited swelling of the hock joints and tendon inflammation. The isolate belonging to genotype II possesses stable replication and clear association with clinical tenosynovitis observed during the originating outbreak.

A commercial inactivated FAdV-4 vaccine was obtained from Zhaoqing Dahuanong Biological Pharmaceutical Co., Ltd. (Zhaoqing, Guangdong, China) and administered according to the manufacturer’s recommended dose and route.

### 2.3. Sample Collection and ARV Detection

Between July 2022 and December 2023, a total of 958 clinical samples were collected from 139 commercial broiler chicken flocks in Fujian, Guangdong, and Guangxi Provinces, China. Flocks exhibited clinical signs consistent with viral arthritis/tenosynovitis, growth retardation, or suspected immunosuppression. Tissue samples (tendon, liver, and cecal tonsil) were aseptically collected from diseased birds. The origin, clinical presentation, and molecular characteristics of the 16 ARV field isolates are summarized in Table 1.

Tissues were homogenized in phosphate-buffered saline (PBS, pH 7.4) to prepare 20% (*w*/*v*) suspensions, subjected to three freeze–thaw cycles, and clarified by centrifugation at 6000× *g* for 5 min at 4 °C. The supernatants were used for virus isolation and nucleic acid extraction. Viral DNA and RNA were extracted using the MagaBio Plus Viral DNA/RNA Purification Kit (Bioer, Hangzhou, China) according to the manufacturer’s instructions. ARV detection was performed using real-time PCR targeting *M1* gene, as previously described [31].

### 2.4. ARV Isolation and Titration in LMH Cells

ARV-positive samples were selected for virus isolation using LMH cells (ATCC CRL-2117, Manassas, VA, USA). Cells were maintained in Dulbecco’s modified Eagle’s medium (DMEM; HyClone, Logan, UT, USA) supplemented with 10% fetal bovine serum (FBS; Bovogen, Keilor East, VIC, Australia) at 37 °C in a humidified atmosphere containing 5% CO_2_.

Clarified supernatants were filtered (0.22 μm) and inoculated onto LMH cell monolayers in 12-well plates. After 1 h adsorption, the inoculum was removed, cells were washed three times using PBS, and maintenance medium containing 2% FBS was added. After 72 h incubation, cells and supernatants were harvested by three freeze–thaw cycles and passaged further. After three blind passages, viral stocks were harvested and subjected to a single additional amplification passage to obtain sufficient viral titers for downstream applications. Therefore, the ARV isolate used for molecular characterization and all animal experiments corresponded to passage 4 (P4) in LMH cells. To evaluate genetic stability during in vitro propagation, the *σC* gene sequence obtained after P4 was compared with that directly amplified from the original clinical specimen, revealing complete nucleotide identity without detectable mutations within the amplified region. These results indicate that no observable sequence variation occurred during limited cell culture passage. Viral titers were determined as TCID_50_/mL as described previously [31].

### 2.5. Molecular Characterization and Phylogenetic Analysis

The full-length *σC* gene was amplified from representative ARV isolates. Purified PCR products were cloned into the pMD19-T vector (TaKaRa, Tokyo, Japan) and transformed into Escherichia coli DH5α competent cells. At least three positive clones per amplicon were sequenced commercially (Sangon Biotech, Guangzhou, China). All nucleotide sequences generated in this study have been deposited in GenBank.

Nucleotide sequence identities were determined using 16 ARV reference strains (Table 2) using the EditSeq and MegAlign programs in DNASTAR Lasergene software (version 7.1; DNAStar, Madison, WI, USA). Multiple sequence alignment was performed using ClustalW, and phylogenetic trees were constructed using the neighbor-joining method with the Maximum Composite Likelihood (MCL) model and 1000 bootstrap replicates in MEGA7.0. Genetic relationships among 16 field isolates and 16 reference strains were analyzed at the nucleotide and amino acid levels.

Phylogenetic analysis based on the *σC* gene was employed as a practical and widely used molecular marker for preliminary epidemiological characterization. Given the segmented nature of the ARV genome and the frequent occurrence of reassortment, σC-based analysis does not fully reflect whole-genome relatedness. Whole-genome sequencing was not performed in the present study due to the large number of field samples, variable viral loads, and the primary focus on integrating molecular surveillance with controlled vaccination-challenge experiments. Future studies incorporating full-genome sequencing will be required to further resolve reassortment patterns and antigenic relationships among circulating ARV strains.

### 2.6. Experimental Model 1: ARV Pathogenicity Assessment

To assess embryonic pathogenicity, twenty 5-day-old SPF embryos were randomly assigned to an infection or a control group (10 embryos per group). Embryos in the infection group were inoculated via the yolk sac with 0.2 mL of ARV GX-742-2022 (10^5^ TCID_50_ per embryo), while controls received an equal volume of sterile saline. Embryos were incubated at 37 °C for 5 days, and those dying within 24 h post-inoculation were excluded. Embryonic growth retardation and gross lesions were recorded at necropsy, and yolk sac membranes and allantoic fluids were collected. Embryonated eggs were observed for 5 days post-inoculation. This endpoint was selected based on preliminary experiments indicating that ARV-associated embryo mortality and characteristic lesions occurred predominantly within the first 3–5 days, with minimal additional informative outcomes beyond this period.

To evaluate the pathogenicity in chickens, forty 5-day-old healthy 818 broilers (a susceptible breed) were randomly divided into an infection or a control group (20 chickens per group). Prior to the experiment, all chickens were verified as seronegative for major avian pathogens, including avian influenza virus, Newcastle disease virus, infectious bursal disease virus, and avian leukosis virus. Chickens in the infection group were inoculated via footpad injection with 0.1 mL of ARV isolate (10^5^ TCID_50_), whereas controls received PBS. Birds were monitored daily for 28 days for clinical signs, body weight, and mortality. At 28 days post-infection (dpi), all birds were euthanized, and gastrocnemius tendons were collected, and fixed in 10% neutral-buffered formalin, and processed for histopathological examination. The footpad inoculation route and early age were selected to establish a synchronized and reproducible infection model for biological interference assessment rather than to replicate natural field transmission dynamics.

### 2.7. Experimental Model 2: ARV-FAdV-4 Infection–Vaccination–Challenge Model

SPF chickens lacking maternal antibodies were used to minimize confounding immune background effects and to allow clear interpretation of ARV-mediated interference with subsequent vaccination. To assess whether prior ARV infection interferes with FAdV-4 vaccine-induced immunity and protection, forty 5-day-old SPF chickens were randomly allocated into four experimental groups (*n* = 10 per group). Group I (ARV + FAdV-4 vaccine + FAdV-4 challenge group): chickens were inoculated with ARV at 5 days of age, vaccinated with the inactivated FAdV-4 vaccine at 14 days of age, and challenged with virulent FAdV-4 at 28 days of age. Group II (FAdV-4 vaccine + FAdV-4 challenge group): chickens were vaccinated at 14 days of age and challenged at 28 days of age. Group III (FAdV-4 challenge group): chickens were challenged with FAdV-4 at 28 days of age without vaccination. Group IV (control group): chickens received PBS only and were neither vaccinated nor challenged. Each bird received 0.2 mL of the corresponding virus suspension. Infection and challenge doses were selected based on preliminary dose-optimization experiments and published studies to ensure reproducible infection while avoiding excessive mortality.

Blood samples were collected from vaccinated birds at 7, 14, and 21 days post vaccination. Serum samples were tested for FAdV-4-specific antibodies using a commercial ELISA kit (Tianjin Speerise Challenge Biotechnology Co., Ltd., Tianjin, China) coated with purified FAdV-4 fiber-2 antigen, following the manufacturer’s protocol. At 36 days of age (21 days post vaccination), birds in groups I, II, and III were challenged intramuscularly with FAdV-4 strain HN1605 (10^6^ TCID_50_). Birds were monitored for 7 days post-challenge for clinical signs and survival, after which all surviving chickens were humanely euthanized. Gross lesions of the liver and heart were recorded. Tissues were fixed in 10% neutral-buffered formalin for histopathological examination. FAdV-4 viral loads in liver tissues were quantified by qPCR as previously described [26]. Viral genome copy numbers were log_10_-transformed prior to analysis and expressed as log10 genome copies per mL of tissue homogenate.

### 2.8. Histopathological Lesion Scoring

Gross and histopathological lesions in the heart and liver following FAdV-4 challenge were evaluated in a blinded manner and scored using a semi-quantitative system. For gross lesions: 0, no lesions; 1, mild hydropericardium (trace fluid) and/or slight hepatic discoloration; 2, moderate hydropericardium (clear fluid distending the sac) with evident hepatomegaly and discoloration; 3, severe hydropericardium with marked hepatomegaly and extensive hepatic changes. For histopathological lesions (H&E staining): 0, within normal limits; 1, mild, multifocal necrosis and inflammation; 2, moderate, multifocal to coalescing necrosis with prominent inflammatory infiltration; 3, severe, diffuse necrosis and massive inflammatory cell infiltration. The final score for each organ per bird represented the consensus of two independent observers. Body weights and liver-to-body weight ratios were not systematically recorded during the terminal necropsy for this specific experiment; therefore, the assessment of hepatomegaly relied on gross morphological evaluation.

### 2.9. Statistical Analysis

All data were collected and analyzed using GraphPad Prism software (version 9.0; GraphPad Software, San Diego, CA, USA). Data normality was assessed using the Shapiro–Wilk test. Normally distributed data were analyzed by one-way analysis of variance (ANOVA), followed by Tukey’s multiple-comparison post hoc test. Non-normally distributed data were analyzed using the Kruskal–Wallis test followed by Dunn’s multiple-comparison test.

Survival differences among groups were evaluated using Kaplan–Meier survival analysis with log-rank (Mantel–Cox) testing. Viral load data were log_10_-transformed prior to statistical analysis. Results are presented as mean ± standard deviation (SD), and differences were considered statistically significant at *p <* 0.05.

## 3. Results

### 3.1. Prevalence and Epidemiological Characteristics of ARV in Commercial Poultry Flocks

**Figure 1:**
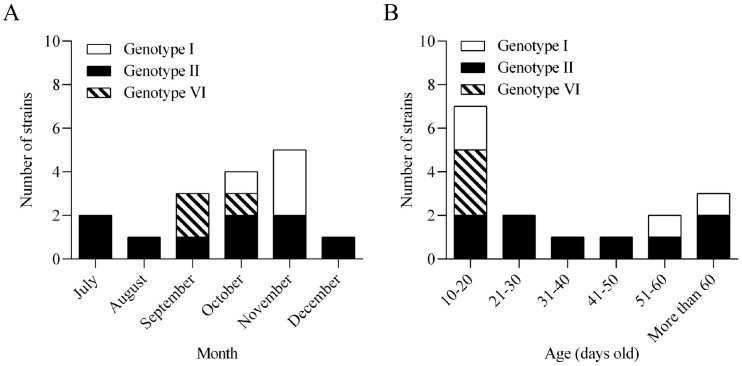
Epidemiological characteristics of ARV isolates from commercial poultry flocks in southern China (July 2022–December 2023). (**A**) Temporal distribution of ARV detection during the study period. ARV isolates were detected throughout the year without a statistically significant seasonal clustering pattern. (**B**) Age distribution of ARV-positive birds, showing that most isolates were obtained from chickens aged 10–20 days.

Between July 2022 and December 2023, a total of 958 clinical samples were collected from 139 commercial broiler and breeder flocks in Fujian, Guangdong, and Guangxi Provinces, China. The sampled flocks exhibited clinical signs consistent with viral arthritis/tenosynovitis, growth retardation, or immunosuppression. Sixteen ARV strains were successfully isolated from these samples, confirming the ongoing circulation of ARV in commercial poultry flocks within the investigated regions. As shown in Figure 1, ARV-positive isolates were detected across different age groups and sampling periods. Guangdong Province accounted for the highest number of isolates, followed by Fujian and Guangxi Provinces. Genotyping based on the *σC* gene demonstrated that genotype II was the predominant genotype circulating in these regions (Table 1). No statistically significant seasonal clustering was detected (*p* > 0.05) (Figure 1A). Age distribution analysis showed that ARV infection occurred across a broad range of ages; however, the highest isolation rate was observed in birds aged 10–20 days (Figure 1B). The frequency of virus isolation declined markedly in older birds. Consistent with previous reports, ARV infection was most frequently detected in young birds, particularly those aged 10–20 days. This age-related distribution reflects the well-recognized susceptibility of early life stages to ARV infection under field conditions.

### 3.2. Virus Isolation, Identification, and Pathogenicity Assessment in SPF Chicken Embryos

**Figure 2:**
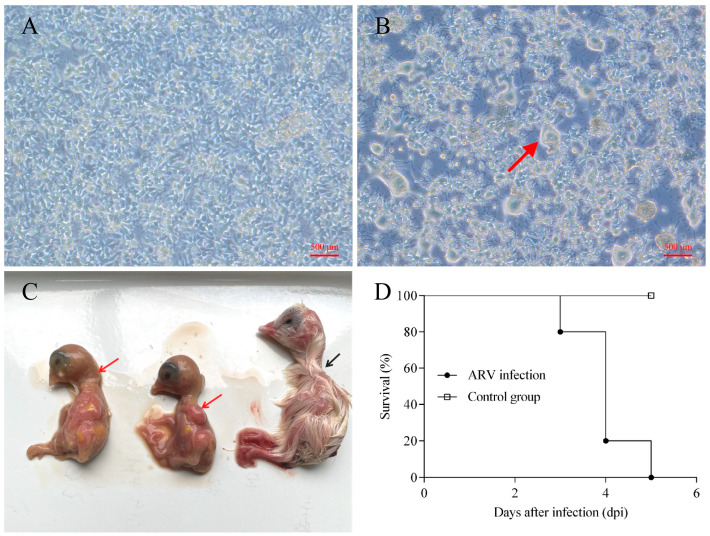
Isolation, in vitro characterization, and in ovo pathogenicity of the ARV strain GX-742-2022. (**A**) Normal morphology of mock-infected LMH cells (control). (**B**) CPE induced by ARV strain GX-742-2022 in LMH cells at 48 h post-infection (hpi), characterized by the formation of multinucleated syncytia (red arrow). (**C**) Gross appearance of 5-day-old SPF chicken embryos at 5 days post-inoculation (dpi). Infected embryos exhibit growth retardation, generalized hyperemia, and hemorrhages in the head and legs (red arrows), whereas control embryos appear normal (black arrow). (**D**) Survival curves of SPF chicken embryos following yolk sac inoculation with ARV GX-742-2022 (10^5^ TCID_50_ per embryo). Mortality began at 3 dpi and reached 100% by 5 dpi in the infected group, while all control embryos survived throughout the observation period (5 days). Embryos that died within 24 h post-inoculation were excluded.

The representative ARV strain GX-742-2022 was successfully isolated through serial passage in Leghorn male hepatoma (LMH) cells. Mock-infected LMH cells maintained normal morphology throughout the observation period (Figure 2A). In contrast, ARV-infected cells exhibited pronounced cytopathic effects (CPE), characterized by the formation of multinucleated syncytia of variable sizes, which became evident at 48 h post-infection following three serial passages (Figure 2B). Successful viral replication was further confirmed by detection of ARV-specific RNA in infected cell cultures using real-time RT-PCR. The isolate used for subsequent experiments corresponded to passage 4 (P4) in LMH cells. Sequencing analysis of the *σC* gene after propagation confirmed complete nucleotide identity with the original clinical sample, indicating genetic stability during limited in vitro passage.

To evaluate pathogenicity in ovo, 5-day-old SPF chicken embryos were inoculated with GX-742-2022 via the yolk sac at a dose of 10^5^ TCID_50_ per embryo. Necropsy revealed marked growth retardation, generalized hyperemia, and hemorrhages in the head and legs of infected embryos (Figure 2C, red arrows), while control embryos displayed normal development (Figure 2C, black arrow). Mortality was first observed at 3 days post-inoculation (dpi), and all infected embryos died by 5 dpi, whereas all embryos in the control group survived throughout the experimental period (Figure 2D).

ARV was successfully re-isolated from embryonic tissues by homogenization and re-inoculation into SPF embryos. RT-PCR amplification yielded the expected 1088-bp fragment, and sequence analysis confirmed 100% nucleotide identity with the original field isolate, fulfilling Koch’s postulates for GX-742-2022.

### 3.3. Genetic Diversity and Phylogenetic Analysis of Circulating ARV Strains

To characterize the genetic diversity of circulating ARV strains, full-length *σC* gene sequences were amplified, cloned, and sequenced from the 16 field isolates. All sequences obtained in this study were deposited in the GenBank database (accession numbers listed in Table 1). Comparative sequence analysis revealed substantial genetic heterogeneity among the isolates, with pairwise nucleotide identities ranging from 57.4% to 98.5%. When compared with 16 representative reference strains retrieved from GenBank, nucleotide identities ranged from 51.1% to 98.0%. The GX-742-2022 strain shared 71.4–98.3% nucleotide identity with genotype II reference strains, indicating marked intra-genotypic variability.

**Figure 3:**
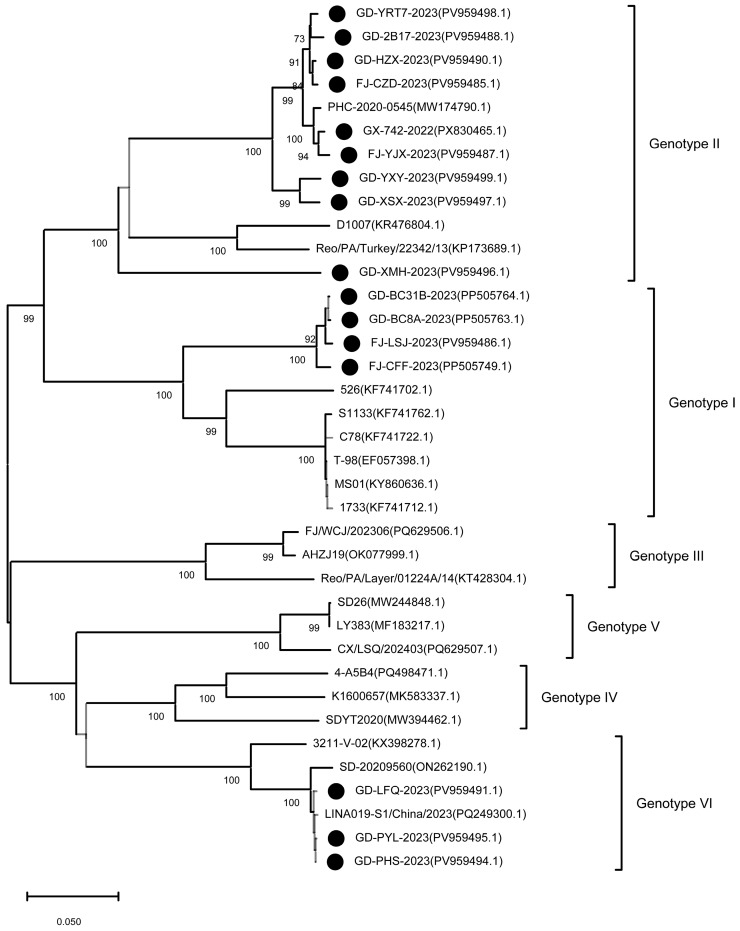
Phylogenetic analysis of avian reovirus based on the *σC* gene. A neighbor-joining phylogenetic tree was constructed using full-length *σC* gene sequences from 16 field isolates (indicated by black circles) and 16 reference strains retrieved from GenBank (labeled with accession numbers). The field isolates clustered into three major genotypes: four isolates in genotype I, nine isolates in genotype II, and three isolates in genotype VI. The scale bar represents nucleotide substitutions per site. Bootstrap values ≥ 70% are shown at branch nodes; values < 70% are indicated in gray lines.

Phylogenetic analysis based on *σC* gene sequences classified the 16 field isolates into three genotypes (Figure 3). Four isolates clustered within genotype I, nine within genotype II, and three within genotype VI. No isolates were assigned to genotypes III, IV, or V. The predominance of genotype II indicates its dominant circulation during the study period. Notably, σC-based analysis revealed clear genetic divergence between circulating field strains and classical vaccine-related strains at the *σC* gene level. Given the segmented nature of the ARV genome and the frequent occurrence of reassortment, these findings should be interpreted as molecular marker-based observations rather than comprehensive whole-genome evolutionary relationships.

### 3.4. Pathogenicity of ARV Strain GX-742-2022 in Broiler Chickens

**Figure 4:**
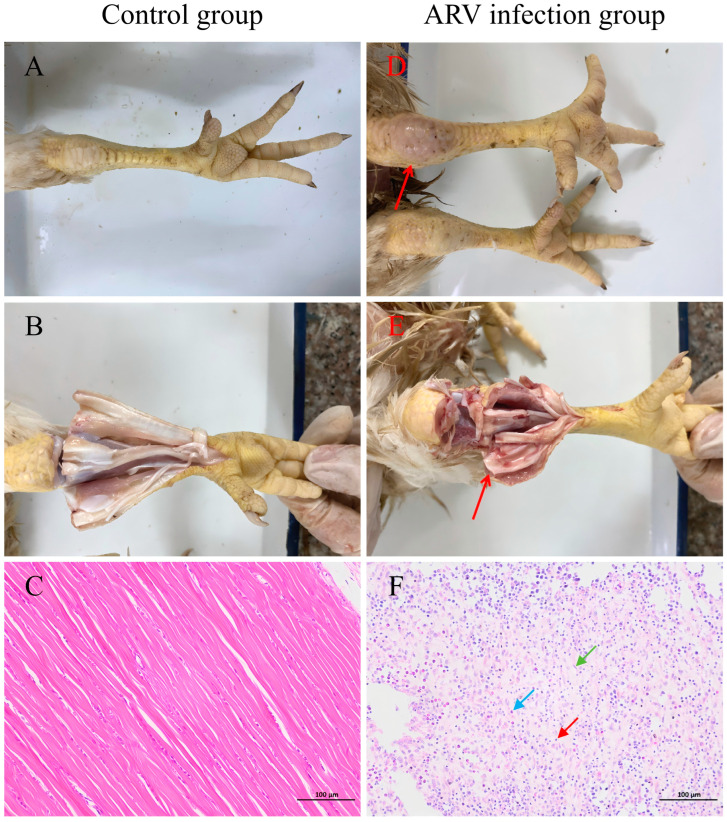
Gross and histopathological lesions induced by avian reovirus strain GX-742-2022 in broiler chickens, and representative images collected at 28 days post-infection. (**A**) Normal external appearance of the tibiotarsal (hock) joint in a control chicken. (**B**) Absence of hemorrhage within the joint cavity upon dissection of a control chicken. (**C**) Histological section of the gastrocnemius tendon from a control chicken showing well-organized, uniformly aligned collagen fibers without necrosis or inflammatory infiltration (H&E staining). (**D**) Pronounced swelling of the tibiotarsal joint in an ARV-infected chicken (red arrow). (**E**) Marked hemorrhage within the joint cavity upon dissection of an ARV-infected chicken (red arrow). (**F**) Histopathological section of the gastrocnemius tendon from an ARV-infected chicken showing severe structural disruption (H&E staining), including focal fragmentation and necrosis of the tendon matrix. Connective tissue hyperplasia is indicated by the green arrow, inflammatory infiltration dominated by lymphocytes and macrophages by the red arrow, and the presence of granulocytes by the blue arrow.

Based on its epidemiological predominance, genetic representativeness, and confirmed pathogenicity in embryos, the genotype II ARV strain GX-742-2022 was selected for further pathogenicity evaluation in broiler chickens. The pathogenicity of ARV strain GX-742-2022 was further evaluated in 5-day-old broiler chickens. At 28 days post-infection (dpi), all birds were euthanized and subjected to necropsy. No gross abnormalities were observed in visceral organs, including the heart, liver, spleen, lungs, or kidneys. In contrast, consistent and pronounced pathological changes were observed in the musculoskeletal system, particularly in the tibiotarsal (hock) joints. Control birds showed normal joint appearance without external swelling (Figure 4A) or internal hemorrhage (Figure 4B). Tendons from control chickens exhibited well-organized connective tissue with uniformly aligned collagen fibers and no evidence of necrosis or inflammatory infiltration (Figure 4C). Infected chickens exhibited marked swelling of the tibiotarsal joints (Figure 4D, red arrow). Upon dissection, hemorrhage within the joint cavity was evident (Figure 4E, red arrow). Histopathological examination of gastrocnemius tendons revealed striking differences between groups. In contrast, tendons from ARV-infected chickens displayed marked structural disruption, characterized by focal fragmentation and necrosis of the tendon matrix (Figure 4F). Prominent reparative responses, including connective tissue hyperplasia (green arrow) and disorganized proliferation of fibroblasts, fibrocytes, and collagen fibers, were observed. These lesions were accompanied by substantial inflammatory infiltration dominated by lymphocytes and macrophages (red arrow), with fewer granulocytes also present (blue arrow). Collectively, these findings demonstrate that GX-742-2022 infection preferentially targets the musculoskeletal system and induces tenosynovitis characterized by joint swelling, hemorrhage, and tendon structural alterations.

### 3.5. Effects of ARV Infection on FAdV-4 Vaccine–Induced Immunity and Protection

#### 3.5.1. Humoral Immune Response

**Figure 5:**
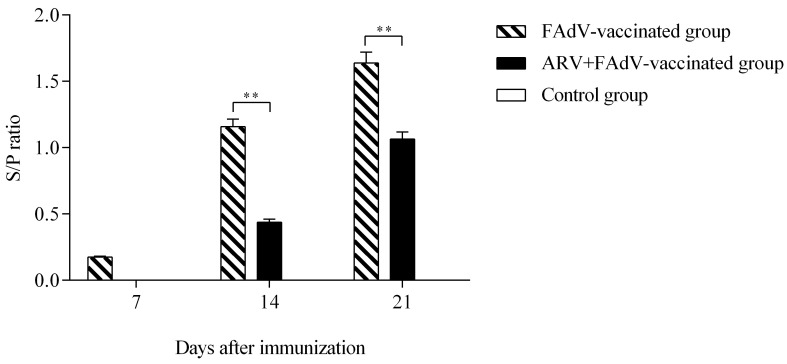
Effect of ARV pre-infection on the humoral immune response to FAdV-4 vaccination. Serum FAdV-4–specific antibody levels were measured by ELISA at 7, 14, and 21 days post-vaccination (dpv) in SPF chickens. All challenged groups were inoculated with FAdV-4 at 28 days of age, as described in experimental model 2. Chickens in the FAdV-4–vaccinated group developed progressively increasing antibody responses over time. In contrast, chickens pre-infected with ARV exhibited significantly lower antibody levels at 14 and 21 dpv compared with the vaccinated-only group (** *p* < 0.01). ELISA results are expressed as optical density (OD) values measured at 450 nm. Data are presented as mean ± SD.

FAdV-4–specific antibody responses were measured at 7, 14, and 21 days following vaccination. Chickens vaccinated with the inactivated FAdV-4 vaccine developed progressively increasing antibody responses over time. In contrast, chickens pre-infected with ARV exhibited significantly lower antibody levels at 14 and 21 days post-vaccination (dpv) compared with the vaccinated-only group (*p <* 0.01) (Figure 5). These results indicate that prior ARV infection is associated with reduced FAdV-4 vaccine-induced humoral responses.

#### 3.5.2. Protective Efficacy and Mortality

**Figure 6:**
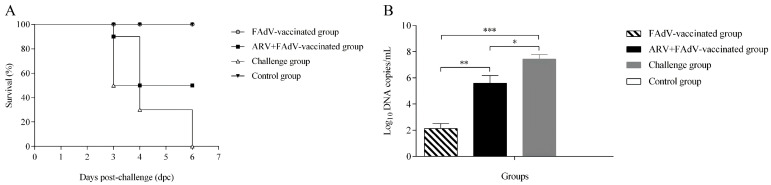
Effect of ARV pre-infection on the protective efficacy of FAdV-4 vaccination and viral replication following challenge. (**A**) Cumulative mortality of chickens following challenge with a virulent FAdV-4 strain. Chickens in the FAdV-4 challenge group exhibited 100% mortality by 6 days post-challenge (dpc). In the ARV + FAdV-4 vaccine + FAdV-4 challenge group, cumulative mortality reached 50% by 7 dpc, whereas no mortality was observed in the FAdV-4 vaccine + FAdV-4 challenge group. No deaths occurred in the control group. Statistical significance between survival curves was determined using the log-rank (Mantel-Cox) test. (**B**) Hepatic viral loads of FAdV-4 at 7 dpc. Viral genome copy numbers in liver tissues were quantified by quantitative PCR (qPCR), and log_10_-transformed prior to statistical analysis. Chickens in the ARV + FAdV-4 vaccine + FAdV-4 challenge group exhibited significantly higher viral loads than the FAdV-4 vaccine + FAdV-4 challenge group (* *p* < 0.05, ** *p* < 0.01, *** *p* < 0.001). Data are presented as mean ± SD.

Following challenge with a virulent FAdV-4 strain, mortality was monitored for 7 days. The FAdV-4 challenge group exhibited 100% cumulative mortality by 6 days post-challenge (dpc). In the ARV + FAdV-4 vaccine + FAdV-4 challenge group, cumulative mortality reached 50% by 7 dpc. In contrast, no mortality was observed in the FAdV-4 vaccine + FAdV-4 challenge group, demonstrating complete protection, while no deaths occurred in the control group (Figure 6A). Kaplan–Meier survival analysis with log-rank testing revealed statistically significant differences among the indicated groups (*p <* 0.05).

#### 3.5.3. Viral Load in Target Organs

To assess peak viral replication, FAdV-4 loads in liver were quantified at 7 days post-challenge (dpc), a time point previously shown to correspond with maximal viral titers in this model. FAdV-4 viral loads in liver tissues were quantified at 7 dpc. Viral genome copy numbers were log_10_-transformed prior to analysis. High viral loads were detected in the challenge-only group. Notably, the ARV + FAdV-4 vaccine + FAdV-4 challenge group exhibited significantly higher hepatic viral loads than the FAdV-4 vaccine + FAdV-4 challenge group (*p <* 0.01) (Figure 6B), indicating enhanced viral replication in the presence of prior ARV infection. The 7 dpc time point was selected based on preliminary experiments indicating peak hepatic viral replication at this stage.

#### 3.5.4. Gross and Histopathological Lesions

Gross and histopathological changes in the heart and liver were evaluated in a blinded manner using a predefined semi-quantitative scoring system, and the distribution of lesion scores is summarized in Table 3. No macroscopic or microscopic lesions were detected in either the control group or the FAdV-4 vaccine plus challenge group. All birds in these two groups received scores of 0 for both organs, consistent with the absence of clinical signs and complete survival. In contrast, chickens in the ARV + FAdV-4 vaccine + FAdV-4 challenge group developed evident but moderate lesions. Gross cardiac lesions were distributed across scores 1–3 (0/3/4/3), with a mean score of 2.0 ± 0.82, whereas hepatic gross lesions were generally milder (1/4/3/2; mean 1.6 ± 0.97). Histopathological damage was more pronounced, with myocardial lesions scored 1/2/4/3 (mean 1.9 ± 0.99) and hepatic lesions 0/2/4/4 (mean 2.2 ± 0.79), characterized by multifocal necrosis and inflammatory cell infiltration. All lesion parameters in this group were significantly higher than those in the vaccinated-and-challenged group (*p <* 0.05).

The FAdV-4 challenge group exhibited the most severe pathological outcome. Typical hydropericardium and hepatomegaly were consistently observed at necropsy, and gross lesion scores were concentrated at grades 2–3 (heart: 0/0/5/5; liver: 0/0/3/7). Histopathological lesions were uniformly severe, with scores of 2–3 in all birds (heart: 0/0/4/6; liver: 0/0/2/8), yielding the highest mean scores among groups (*p <* 0.05). Compared with this group, lesion severity in the ARV-preinfected group was reduced for several parameters but remained significantly higher than in fully protected birds, indicating incomplete protection.

**Figure 7:**
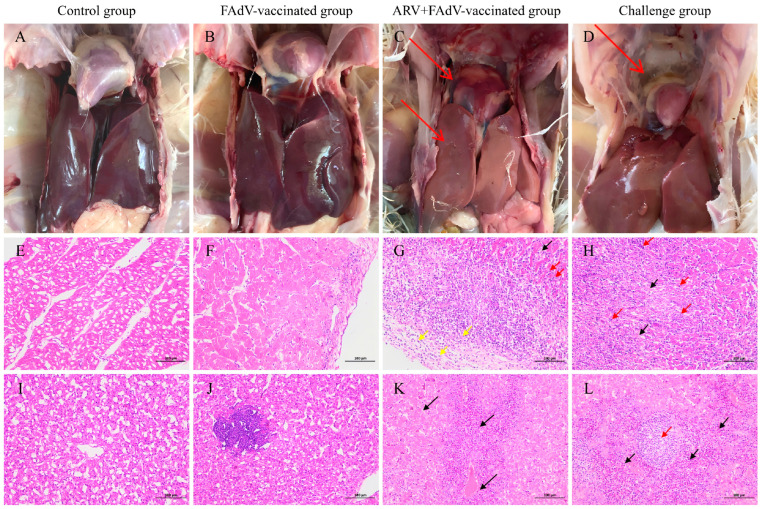
Gross and histopathological lesions in chickens following FAdV-4 challenge, illustrating the impact of prior ARV infection on vaccine-mediated protection. (**A**) Normal gross appearance of the heart and liver in a control chicken. (**B**) Heart and liver from a chicken in the FAdV-4 vaccine + FAdV-4 challenge group, showing no apparent gross lesions. (**C**) Gross lesions in a chicken from the ARV + FAdV-4 vaccine + FAdV-4 challenge group, characterized by hepatomegaly, hepatic discoloration, and hydropericardium (red arrows). (**D**) Mild to moderate hydropericardium observed in a chicken from the FAdV-4 challenge group (red arrow). The fluid accumulation observed in the pericardial sac was defined as hydropericardium based on standardized gross examination criteria, including transparent fluid accumulation without evidence of post-mortem hemorrhage. (**E**) Normal histological architecture of cardiac tissues from a control chicken (H&E staining). (**F**) Normal cardiac tissue from a chicken in the FAdV-4 vaccine + FAdV-4 challenge group (H&E staining). (**G**) Cardiac tissue from a chicken in the ARV + FAdV-4 vaccine + FAdV-4 challenge group. Marked myocardial necrosis (black arrow) with focal infiltration of lymphocytes and macrophages (red arrows) and diffuse inflammatory cell infiltration (yellow arrows) (H&E staining). (**H**) Cardiac tissue from a chicken in the FAdV-4 challenge group. Loss of normal myocardial architecture with marked cardiomyocyte necrosis (black arrows) and prominent focal infiltration of lymphocytes and macrophages (red arrows) (H&E staining). (**I**) Normal histological architecture of hepatic tissue from a control chicken. (**J**) Normal hepatic tissue from a chicken in the FAdV-4 vaccine + FAdV-4 challenge group. (**K**) Hepatic tissue from a chicken in the ARV + FAdV-4 vaccine + FAdV-4 challenge group. Multiple foci of hepatocellular necrosis (black arrows) (H&E staining). (**L**) Hepatic tissue from a chicken in the FAdV-4 challenge group. Numerous foci of hepatocellular necrosis (black arrows) accompanied by focal lymphocytic infiltration (red arrow) (H&E staining).

Gross findings were consistent with the scoring results. No visible lesions were detected in the vaccinated-and-challenged group or in controls (Figure 7A,B). Hydropericardium and hepatic enlargement were evident in the ARV + FAdV-4 vaccine + FAdV-4 challenge group (Figure 7C), and the challenge group (Figure 7D). Histologically, myocardial necrosis with inflammatory infiltration and multifocal hepatocellular necrosis were observed in the ARV + FAdV-4 vaccine + FAdV-4 challenge group, whereas more extensive and diffuse lesions were present in the FAdV-4 challenge group. Cardiac and hepatic tissues from vaccinated and control birds maintained normal architecture (Figure 7E–L).

Collectively, these quantitative and semi-quantitative data demonstrate that prior ARV infection was associated with aggravated pathological damage following FAdV-4 challenge, consistent with the reduced vaccine-mediated protection observed in this study.

## 4. Discussion

ARV has long been recognized as a ubiquitous poultry pathogen [2,32]. However, its broader role as a virus associated with modulation of vaccine responsiveness with vaccine-induced protection remains insufficiently characterized. By integrating field surveillance, molecular epidemiology, and a controlled infection–vaccination–challenge model, this study demonstrates that prior ARV infection is associated with impaired vaccine-induced humoral responses and reduced protective efficacy against *Aviadenovirus hydropericardii* (FAdV-4).

Our epidemiological investigation revealed widespread ARV circulation in broiler chickens and breeder flocks across multiple provinces, including in flocks without overt clinical disease. This is consistent with recent surveillance reporting frequent ARV detection in clinically healthy birds, highlighting its capacity for subclinical or persistent infection [2,33,34,35]. Such silent circulation likely facilitates sustained transmission and complicates disease control [2,36].

Phylogenetic analysis of *σC* gene demonstrated pronounced genetic divergence between contemporary field isolates and classical vaccine strains, with genotype II predominating. Similar patterns of rapid ARV evolution and genotype replacement have been reported in multiple regions worldwide [9,34,37]. Given the critical role of σC protein in host cell attachment and neutralizing antibody induction, its genetic variation likely contributes to reduced cross-protection and persistent circulation despite vaccination [36,37,38,39]. However, due to the segmented genome of ARV and the frequent occurrence of reassortment, σC-based divergence should be interpreted as a molecular marker-based observation rather than a comprehensive representation of whole-genome evolutionary or antigenic relationships. In this context, σC genotyping serves as a practical epidemiological surveillance tool rather than a substitute for full-genome analysis.

Beyond its epidemiological significance, our experimental data demonstrate that ARV infection induces characteristic immunopathological changes. Infected chickens developed severe tenosynovitis and tendon degeneration with inflammatory infiltration, consistent with classical ARV-associated viral arthritis [33,36,40]. Importantly, these lesions occurred without pronounced systemic pathology, suggesting that ARV-mediated immune dysfunction may persist without dramatic mortality, thereby remaining underrecognized in commercial settings [6,8,36].

A key experimental observation of this study is that prior ARV infection compromises the immunogenicity and protective efficacy of an inactivated FAdV-4 vaccine under controlled experimental conditions. Chickens infected with ARV before vaccination exhibited reduced FAdV-4-specific antibody responses, decreased survival following challenge, increased hepatic viral loads, and aggravated gross and histopathological lesions. Importantly, the novelty of this work does not lie in proposing a previously unrecognized interaction between ARV and FAdV-4, as synergistic effects among immunosuppressive viruses have been reported previously. Rather, the present study provides quantitative experimental evidence, using a defined infection–vaccination–challenge interference model, that ARV infection directionally impairs vaccine-induced protection against an unrelated but economically important pathogen. This phenomenon parallels observations from other immunosuppressive disease models, including variant IBDV, which has been shown to reduce FAdV-4 vaccine efficacy and enhance disease severity [26,27,28,41].

It is important to emphasize that the experimental model employed in this study was designed to demonstrate biological plausibility and directionality of ARV-mediated vaccine interference rather than to fully recapitulate natural field exposure conditions. The use of young SPF chickens without maternal antibodies and footpad inoculation ensured synchronized infection and a controlled immune background, enabling precise assessment of interference effects. Although this approach does not reflect the complexity of commercial production environments, it provides a robust framework for mechanistic evaluation and facilitates interpretation of field observations.

The mechanisms underlying ARV-associated impairment of vaccine responsiveness are likely multifactorial. Previous studies have demonstrated that ARV infection can damage lymphoid tissues, alter cytokine and interferon signaling, and interfere with antigen presentation, collectively impairing the development of effective adaptive immune responses [19,42,43,44,45]. Although the present study did not directly assess these mechanisms, the consistent reduction in vaccine-induced antibody responses, coupled with increased viral replication and disease severity, indicates that ARV infection creates an immunological environment unfavorable for optimal vaccine responsiveness [14,46].

It should be noted that immune impairment in this study was primarily evaluated at the level of humoral responses using ELISA-based antibody measurements. Comprehensive immunological parameters, including lymphoid organ indices, cellular immune profiling, cytokine responses, or virus-neutralizing antibody assays, were not assessed. Therefore, the observed effects should be interpreted as reduced vaccine responsiveness rather than definitive evidence of systemic immunosuppression. Future studies incorporating cellular immune analyses and functional neutralization assays will be essential to further elucidate the immunological mechanisms underlying ARV-mediated vaccine interference.

From a practical perspective, these findings have important implications for poultry health management. In regions where ARV is ubiquitous, early-life infection may predispose flocks to vaccine failure against FAdV-4 and potentially other pathogens [36].This underscores the need for integrated control strategies that consider the cumulative impact of immunosuppressive agents, rather than relying solely on single-pathogen vaccination. Enhanced biosecurity, optimized vaccination timing, and updated vaccines targeting prevalent ARV genotypes may be required to improve overall vaccine performance [32].

Several limitations warrant consideration. Only one representative ARV strain was evaluated, and strain-specific differences in immunosuppressive capacity cannot be excluded. Moreover, the experimental design does not fully recapitulate commercial production environments, where co-infections and environmental stressors may further modulate immune responses. Different ARV genotypes, infection doses, and infection–vaccination intervals may differentially affect immune interference, warranting further investigation. Future studies incorporating multiple ARV genotypes, co-infection models, and detailed immunological profiling are needed to fully elucidate the mechanisms and field relevance of ARV-mediated immune interference.

In conclusion, this study demonstrates that ARV circulates widely in commercial poultry flocks and can act as a biologically relevant modifier of vaccine-induced protection. Under controlled experimental conditions, prior ARV infection impaired FAdV-4 vaccine-induced antibody responses, reduced protective efficacy, and exacerbated disease outcomes following challenge. These findings highlight the potential risk of early ARV exposure for vaccination outcomes and emphasize the importance of incorporating ARV surveillance and control into integrated poultry disease management programs.

## Figures and Tables

**Table 1:** Origin, clinical presentation, and molecular characteristics of avian reovirus field isolates from southeastern China (2022–2023).

No.	Isolate	Genotype	Accession No.	Region	Collection Date	Age (Days)	Clinical Symptoms	Sample Origin
1	FJ-CFF-2023	I	PP505749.1	Fujian	November, 2023	64	Tenosynovitis	Tendons
2	GD-BC8A-2023	I	PP505763.1	Guangdong	November, 2023	18	Malabsorption	Liver
3	GD-BC31B-2023	I	PP505764.1	Guangdong	November, 2023	12	Tenosynovitis	Cecal tonsil
4	FJ-LSJ-2023	I	PV959486.1	Fujian	October, 2023	56	Malabsorption	Tendons
5	FJ-YJX-2023	II	PV959487.1	Fujian	July, 2023	35	Tenosynovitis	Cecal tonsil
6	FJ-CZD-2023	II	PV959485.1	Fujian	August, 2023	17	Tenosynovitis	Tendons
7	GD-2B17-2023	II	PV959488.1	Guangdong	October, 2023	29	Tenosynovitis	Cecal tonsil
8	GD-HZX-2023	II	PV959490.1	Guangdong	September, 2023	23	Tenosynovitis	Cecal tonsil
9	GD-XMH-2023	II	PV959496.1	Guangdong	October, 2023	89	Tenosynovitis	Cecal tonsil
10	GD-YXY-2023	II	PV959499.1	Guangdong	November, 2023	44	Tenosynovitis	Tendons
11	GD-XSX-2023	II	PV959497.1	Guangdong	November, 2023	19	Tenosynovitis	Tendons
12	GX-742-2022	II	PX830465.1	Guangxi	July, 2022	112	Tenosynovitis	Tendons
13	GD-YRT7-2023	II	PV959498.1	Guangdong	December, 2023	58	Malabsorption	Liver
14	GD-PYL-2023	VI	PV959495.1	Guangdong	October, 2023	11	Tenosynovitis	Tendons
15	GD-LFQ-2023	VI	PV959491.1	Guangdong	September, 2023	16	Tenosynovitis	Tendons
16	GD-PHS-2023	VI	PV959494.1	Guangdong	September, 2023	10	Tenosynovitis	Tendons

**Table 2:** ARV reference strains retrieved from the GenBank database.

No.	Strain	Genotype	Accession No.	Country	Year
1	MS01	I	KY860636.1	China	2013
2	526	I	KF741702.1	China	2013
3	1733	I	KF741712.1	China	2013
4	C78	I	KF741722.1	China	2013
5	S1133	I	KF741762.1	China	2013
6	T-98	I	EF057398.1	China	2006
7	PHC-2020-0545	II	MW174790.1	China	2020
8	Reo-PA-Turkey-22342-13	II	KP173689.1	USA	2013
9	D1007	II	KR476804.1	Hungary	2015
10	AHZJ19	III	OK077999.1	China	2019
11	FJ/WCJ/202306	III	PQ629506.1	China	2023
12	Reo-PA-Layer-01224A-14	III	KT428304.1	USA	2014
13	K1600657	IV	MK583337.1	USA	2016
14	SDYT2020	IV	MW394462.1	China	2020
15	4-A5B4	IV	PQ498471.1	China	2024
16	LY383	V	MF183217.1	China	2017
17	CX/LSQ/202403	V	PQ629507.1	China	2023
18	SD26	V	MW244848.1	China	2019
19	3211-V-02	VI	KX398278.1	Hungary	2002
20	LINA019-S1/China/2023	VI	PQ249300.1	China	2023
21	SD-20209560	VI	ON262190.1	China	2020

**Table 3:** Individual histopathology scores and gross lesion observations in chickens following FAdV-4 challenge.

Group	Heart Gross Score (0/1/2/3, n)	Liver Gross Score (0/1/2/3, n)	Heart Histopathology Score (0/1/2/3, n)	Liver Histopathology Score (0/1/2/3, n)	Mean Heart Gross	Mean Liver Gross	Mean Heart Histopathology	Mean Liver Histopathology
Control group	10/0/0/0	10/0/0/0	10/0/0/0	10/0/0/0	0.0 ± 0.0 ^a^	0.0 ± 0.0 ^a^	0.0 ± 0.0 ^a^	0.0 ± 0.0 ^a^
FAdV-4 vaccine + FAdV-4 challenge group	10/0/0/0	10/0/0/0	10/0/0/0	10/0/0/0	0.0 ± 0.0 ^a^	0.0 ± 0.0 ^a^	0.0 ± 0.0 ^a^	0.0 ± 0.0 ^a^
ARV + FAdV-4 vaccine + FAdV-4 challenge group	0/3/4/3	1/4/3/2	1/2/4/3	0/2/4/4	2.0 ± 0.82 ^b^	1.6 ± 0.97 ^b^	1.9 ± 0.99 ^b^	2.2 ± 0.79 ^b^
FAdV-4 challenge group	0/0/5/5	0/0/3/7	0/0/4/6	0/0/2/8	2.5 ± 0.53 ^b^	2.7 ± 0.48 ^c^	2.6 ± 0.52 ^c^	2.8 ± 0.42 ^c^

Lesions were scored using a semi-quantitative system (0–3) as described in Section 2 . Scores are presented as the number of birds per score category. Values for mean scores are expressed as mean ± SD. Different superscript letters (^a^, ^b^, ^c^) within a column indicate statistically significant differences between groups (*p <* 0.05, Kruskal–Wallis test with Dunn’s post hoc test). Groups sharing the same letter are not significantly different.

## Data Availability

The original contributions presented in this study are included in the article. Further inquiries can be directed to the corresponding authors.
